# The Influence of Diet and Obesity in Lynch Syndrome: What Do We Know So Far

**DOI:** 10.3390/nu16244352

**Published:** 2024-12-17

**Authors:** Cláudio Rodrigues, Susana Couto Irving, Paula Alves, Mário Dinis-Ribeiro, Catarina Brandão, Marta Correia

**Affiliations:** 1 Gastroenterology Department, Unidade Local de Saúde de Viseu Dão Lafões, 3504-509 Viseu, Portugal; claudiomelorodrigues@gmail.com; 2 Nutrition–Medicine Department, Instituto Português de Oncologia, Francisco Gentil E.P.E., 4200-072 Porto, Portugal; smscouto.irving@gmail.com (S.C.I.); palves@ipoporto.min-saude.pt (P.A.); 3 IRISE@CI-IPOP (Health Research Network), Portuguese Oncology Institute of Porto (IPO Porto), 4200-072 Porto, Portugal; mdinisribeiro@gmail.com; 4 MEDCIDS-Department of Community Medicine, Information and Decision in Health, Faculty of Porto, University of Medicine, 4200-072 Porto, Portugal; 5 Department of Gastroenterology, Porto Comprehensive Cancer Center, 4200-072 Porto, Portugal; 6 Laboratório Associado, Escola Superior de Biotecnologia, Centro de Biotecnologia e Química Fina, Universidade Católica Portuguesa, CBQF, Rua Diogo Botelho 1327, 4169-005 Porto, Portugal; mmcorreia@ucp.pt

**Keywords:** Lynch syndrome, diet, colorectal cancer risk, body mass index, body fat

## Abstract

Of all new cases of colorectal cancer, Lynch syndrome (LS) accounts for approximately 3%. This syndrome is the most common hereditary cancer syndrome and is caused by pathogenic variants in the genes responsible for DNA mismatch repair. Although the relationship between colorectal cancer risk and diet is well established, little is known regarding the influence of diet and nutritional characteristics on LS’s clinical evolution. There is some evidence suggesting that individuals living with LS should follow general guidelines for diet and alcohol restriction/moderation, so as to achieve and maintain a favorable weight status and overall health and quality of life. However, more research is needed, preferentially from clinical studies of a prospective nature with robust designs, to better inform diet and behavioral patterns targeting cancer prevention in LS.

## 1. Introduction

It is estimated that more than 1.9 million new colorectal cancer (CRC) cases and approximately one million deaths, which were attributable to this cancer, occurred in 2020 [1]. Of all new cases of CRC, Lynch syndrome (LS) may be accountable for 3% [2]. Lynch syndrome (LS) is the most common hereditary cancer syndrome [3]. This dominantly inherited syndrome is caused by pathogenic variants in the genes responsible for DNA mismatch repair (MMR), i.e., MLH1, MSH2, MSH6, or PMS2, or in the EPCAM gene (leading to the silencing of the MSH2 gene) [4]. The estimated cumulative risk to age 70 years stands between 40% and 70% depending on the carrier’s sex and the mutated MMR gene [5,6]. Due to the diverse penetrance of the MMR mutation seen in families affected by LS and also taking into account the geographic variation in LS-based CRC risk [7], it seems reasonable to expect some influence of environmental and/or lifestyle factors on the genesis of colorectal cancer.

Diet has been one of the most relevant recognized modifiable risk factors in sporadic CRC [8], with a considerable number of studies investigating the association between specific components of diet and the risk of CRC; others highlight the association between a western dietary pattern and CRC [6]. Likewise, modifications in dietary patterns, along with changes in lifestyle factors such as obesity, alcohol, and tobacco consumption, have been advocated to impact the increase in sporadic CRC [8]. In LS, most of the research and reports have concentrated on smoking habits and body fat [9], and only a few studies have tried to address the putative influence of diet [9,10,11,12], providing mixed results [11,12].

In this review, we aim to summarize the current literature regarding the influence of diet and weight status in LS.

## 2. Materials and Methods

**Figure 1:**
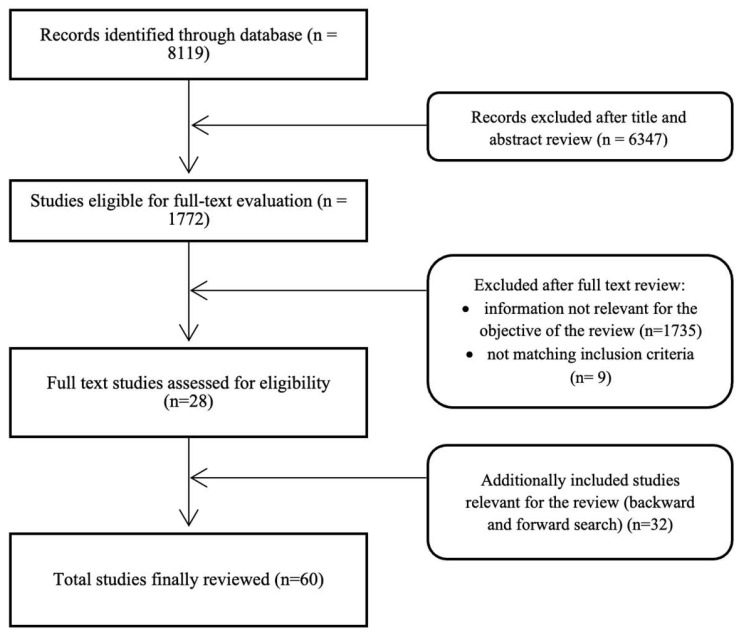
Flow diagram demonstrating the literature search and eligibility.

For this narrative review, PubMed was queried for relevant studies conducted from inception to 30 June 2024, using the following terms: “Lynch syndrome”, “mismatch repair”, “hereditary colorectal cancer”, “MLH1”, “MSH2”, “MSH6”, “PMS2”, “EPCAM”, and “diet” and/or “nutrition”, and/or “obesity”, and/or “weight status” to identify pertinent publications exploring the influence of diet and weight status/obesity in the risk of CRC in patients with Lynch syndrome. The articles included in this review are original retrospective and prospective cohort studies, randomized controlled trials, and systematic reviews/metanalyses; all are written in English. Additionally, a backward search of the references of the identified articles also retrieved some other relevant articles. Similarly, a forward search found recent articles that cited the screened papers. Animal studies were excluded. Figure 1 illustrates the selection process.

## 3. Risk of Colorectal Cancer in People Living with Lynch Syndrome

### 3.1. Influence of Dietary Components and Supplementation on CRC Risk

#### 3.1.1. Dietary Fiber

In the general population, the overall existing evidence is convincing regarding the significant decrease in CRC risk amongst individuals with a higher intake of dietary fiber [8]. In patients with LS, a CAPP-2 secondary analysis study [13] looking at 10-year follow-up for all participating sites assessed the influence of resistant starch (RS) on the incidence of CRC and other LS-related cancers. RS is a component of dietary fiber that is present in foods such as bananas, potatoes, and grains. Its postulated beneficial mechanism of action may be related to its metabolites that are generated by gut bacteria, such as butyrate, which demonstrate anti-inflammatory properties in vitro [14]. In a study by Bansal et al., the quotidian use of RS did not decrease the incidence of colorectal cancer (HR = 0.92; 95% confidence interval (CI), 0.62–1.34; *p* = 0.63) but, interestingly, reduced the incidence of some extracolonic LS cancers, including upper gastrointestinal cancers (stomach, duodenum, pancreas, and bile duct). The mechanism behind this protective effect on upper GI tumors is unknown. Of note, RS did not impact the incidence of cancers not related to this syndrome. Ultimately, it has been suggested that LS individuals should eat around 30 g of RS per day [15].

The World Cancer Research Fund (WCRF)/American Institute for Cancer Research (AICR) Cancer Prevention Recommendations Report advises people to consume wholegrains, as they are a source of dietary fiber, and various vitamins and minerals; substantial evidence shows a decreased risk with an increased consumption of wholegrains [8]. To our best knowledge, this has not been studied so far in relation to LS.

#### 3.1.2. Meat

The consumption of red and processed meat has been associated with an increased risk of sporadic CRC [16]. In the context of LS, the assessment of distinctive eating patterns over a median follow-up period of 20 months [11] when duly adjusted for age and sex, highlighted a significant association of developing colorectal adenoma in those with a pattern of fresh and processed meat intake (tertile 3—HR: 2.48; 95% CI: 1.22, 5.02). However, this association was not significant in fully adjusted models (i.e., age, sex, smoking status, and adenoma or bowel resection status) [11].

A case–control study [17] from the Netherlands included 62 adenoma cases from LS (all families fulfilled the Amsterdam criteria but MMR mutation status was unknown) and 83 adenoma-free controls. Interestingly, the investigators were also able to look into not only red meat and poultry consumption, but also gather details on preparation methods. However, the previous associations between meat intake and adenoma risk did not prevail (OR for high vs. low consumption: 0.6; 95% CI: 0.2–1.6) [17]. When these models were adjusted for age, gender, and, importantly, total calories, alongside meat intake, still no significant association was captured [17].

#### 3.1.3. Dietary Supplementation

Heine-Broring et al. [18] assessed the use of supplements (multivitamin, vitamin C, calcium, or fish oil supplementation) among 470 individuals with LS over a median follow-up time of 39 months. No significant associations between risk of developing colorectal adenoma and the use of any dietary supplement was found when assessed separately or by total fruit and vegetable consumption in adjusted models (age and sex, or age, sex, and number of colonoscopic procedures during follow-up) [18].

Likewise, the cohort study GEOLynch observed no association between colorectal tumor (CRT) risk and dietary vitamin B intake (folate, vitamin B2, vitamin B6, vitamin B12; e.g., HR for highest vs. lowest tertile of vitamin B2: 0.77, 95% CI: 0.39–1.51) or methionine intake (HR for highest vs. lowest tertile: 1.35; 95% CI: 0.83–2.20) [12,19].

Others looked into 1966 individuals with LS and recalled their multivitamin, calcium, and folic acid supplementation in the risk of colorectal cancer in [20]. Overall, a 36% reduction in CRC risk with any past usage of a multivitamin and calcium supplement (HR: 0.36; 95% CI: 0.20–0.64) after adjustments in analysis (namely, gender, nationality, education, frequent physical activity, smoking, non-steroidal anti-inflammatory use, and number of screening colonoscopies). When considering a 3 year supplementation timeframe, a close-to-50% reduced risk of CRC was found compared with never users (multivitamin intake—HR: 0.47; 95% CI: 0.32–0.69; calcium intake—HR: 0.42; 95% CI: 0.23–0.74). No evidence was found for the association between folic acid supplement intake and CRC risk (*p* = 0.82).

The case–control study of Diergaarde et al. [21] did not report any statistical significance regarding dietary supplementation and CRC risk, namely, calcium, vitamin C, beta-carotene, and folic acid, including adjustments for mutation carrier status, sex, age at last colonoscopy, tobacco use, and total energy intake [21].

Elsewhere, calcium intake through supplements seemed to decrease the risk of pre-malignant lesions such as colorectal adenomas [22]. This report aligns with the observed inverse associations between the intake of calcium-rich dairy products, such as milk and cheese, and colorectal cancer development for the general population [8]. In LS, and in the absence of studies specifically exploring the consumption of dairy foods and CRC risk, calcium’s chemoprevention potential builds on the theory that it binds to fatty acids and bile salts, resulting in lower colonic irritation and injury-related cell proliferation [23]. An old placebo-controlled trial where calcium carbonate supplements were used over a 12 week period failed to demonstrate a significant benefit related to the reduction in cell proliferation in the distal colorectum, which was evaluated using biopsy specimens acquired prior to and after the intervention [24].

### 3.2. Influence of Alcohol Consumption

Alcohol is a known carcinogen for the general population. The metabolite acetaldehyde has been implicated as the culprit behind carcinogenesis. It can be found in high levels in the colon following ethanol ingestion [25]. Acetaldehyde interferes with DNA synthesis and repair, affects the function of the anti-oxidant glutathione, and enhances colonic mucosal proliferation [26].

A few studies have explored the association between alcohol consumption and colorectal cancer risk. In the study of Diergaarde et al. [21], no significant associations were reported (OR (95% CI) of 12.8 g/day versus 2.6 g/day, 1.0 (0.5–2.0), and *p* for trend = 0.85) with matching for age at last colonoscopy, sex, total energy intake, carrier status, and cigarette smoking [21]. In a retrospective cohort study of 360 MLH1- or MSH2 mutation carriers with the primary aim of determining whether cigarette smoking would alter CRC risk, alcohol use information was available in 271 carriers, of whom 83 (30.6%) were classified as non-users. Alcohol use did not demonstrate a statistically significant effect on colorectal cancer risk (*p* value > 0.40), and, again, adjusted for MMR mutation, gender, and birth cohort [27]. Using data from the GeoLynch cohort study, a prospective analysis of 386 subjects with LS, during a median follow-up of 10 months, revealed a significant association between adenoma risk and alcohol ingestion only at the highest tertile (13–71 g alcohol per day) within the unadjusted model (HR: 2.24; 95% CI: 1.09–4.60). However, after adjust for smoking status (HR: 1.87; 95% CI: 0.87–3.99) and all factors (age, sex, smoking status, number of colonoscopies, colorectal resection, and BMI), no significant associations were detected [28]. Conversely, Dashti et al. found a positive association between the consumption of alcohol and CRC risk in persons with LS [25]. Irrespective of MMR gene mutation, a nearly twofold increase in CRC risk (HR: 1.79; 95% CI: 1.12, 2.87) was registered when individuals with LS consumed >28 g of alcohol per day. There was no relationship with gender (*p* > 0.05). These studies also took into account the reported BMI at age 20, diabetes status, smoking habits, regular physical activity, education, and country [25]. Similarly, in Japan, those who previously or currently consume alcoholic drinks [29] had an increased risk of CRC (HR: 2.44; 95% CI: 1.13, 5.16) [29]. Another study from Asia, a retrospective cohort study [30] investigating the risk factors associated with the development of CRC in patients with MLH1 and MSH2 germline mutations, observed an association only among MSH2 carriers (MLH1 carriers—HR: 0.73; 95% CI: 0.45–1.16; *p* = 0.185), who had a twofold increase in CRC risk with alcohol consumption (HR: 2.33; 95% CI: 1.04–5.21; *p* = 0.038) [30].

On the basis of the studies presented assessing alcohol intake, the correlation between alcohol intake and CRC risk in patients with LS seems to favor traditional recommendations for the general population [25,30]; as such, individuals with LS should probably abstain from regular alcohol consumption.

### 3.3. Dietary Patterns

Limitations inherent to single food and nutrient studies, as well as the perception of conceivable synergistic effects between foods, have stimulated the use of new approaches to investigate the association between diet and disease. Over the past few years, principal component analysis has emerged as a statistical method to derive dietary patterns aiming to assess the relation between diet and disease, mainly in observational studies [6,11]. This approach allows for the aggregation of foods or food groups that are usually ingested together, thus establishing a dietary pattern [6].

Several epidemiological studies show an influence of dietary patterns on the risk of sporadic colorectal adenomas and cancer [6,31,32]. Namely, Western and Prudent dietary patterns have been associated, respectively, with higher and lower risks of colorectal cancer [33,34,35]. In the prospective study by Botma et al. [11], looking at 486 MMR gene mutation carriers and following the adjustment of the model concerning age, sex, colorectal adenoma history, and extent of colonic resection, the dietary pattern consistent with a high intake of snack foods (chips, fried snacks, fast food snacks, spring rolls, mayonnaise-based sauces, cooking fat and butter, peanut sauce, ketchup, sweets, and diet sodas) emerged as causing a significant increase in colorectal adenoma risk (HR for highest vs. lowest tertile: 2.16; 95% CI: 1.03–4.49). Moreover, in this study, all the dietary information was collected using food frequency questionnaires, which also showed other dietary patterns, such as ‘Meat’ and ‘Cosmopolitan’; however, none of these had statistically significant associations with colorectal adenoma [11].

### 3.4. Inflammatory Potential of the Diet and Diet Quality

In patients with LS, the first study aimed to investigate the association between the inflammatory potential of the diet and CRT risk used a hypothesis-driven nutrient-based index [12]. For that purpose, Brouwer et al. [12] analyzed the dietary intake of 457 participants from the prospective cohort study GEOLynch to calculate an adapted dietary inflammatory index (ADII). This ADII was divided into tertiles, in which the highest tertile mirrors the most pro-inflammatory potential of the diet. Nevertheless, no significant association between the pro-inflammatory potential of the diet and a risk of LS-associated colorectal tumors was observed [12]. Furthermore, it was not possible to determine a difference between the impact of the inflammatory potential of the diet in colorectal adenoma and CRC risk in persons with LS [12]. But, a study caveat stems from the use of a nutrient-based index that does not take into account the fact that foods may have intricate effects on health due to different structures, preparation practices, and nutrient interactions [7].

To overcome the previously stated limitation of a nutrient-based index, the GeoLynch cohort subsequently derived yet another study that was the first to investigate the association between food-based diet quality indexes and colorectal tumors risk in persons living with LS [7]. Similarly, no statistically significant associations were yielded between the Dutch Healthy Diet index 2015 (DHD15-index) or the Dietary Approaches to Stop Hypertension (DASH score) and CRT risk. [7]. The limitation raised is the omission of data on coffee, salt, and sweetened beverage intake, which are recognized as potentially affecting diet quality evaluations. However, a previous meta-analysis combining five studies concerning sporadic CRC showed that individuals classified in the highest category of the DASH score had a decreased CRC risk compared to patients in the lowest category (HR: 0.80; 95% CI: 0.74—0.85), demonstrating that the risk of sporadic CRC is negatively associated with the adherence to the DASH dietary pattern [36]. Hence, associations found in the general population between diet quality and CRC risk might not remain in persons with LS. The contrasting associations found in LS studies versus those referring to the general population could be explained by the different mechanisms behind CRT development, as has been corroborated by studies demonstrating that adenomas in patients with LS are primarily microsatellite instability (MSI)-high [37], whereas sporadic adenomas are mainly MSI-low [38].

Further prospective studies and clinical trials are required to provide additional evidence on how dietary modifications for individuals with LS could be beneficial in reducing CRC in this high-risk setting. Until then, it is important that the Prudent dietary recommendations established for the general population should also be encouraged and followed by LS patients (Table 1).

## 4. Obesity and the Risk of Colorectal Cancer in People Living with Lynch Syndrome

The relationship between obesity and cancer risk has been a focus of extensive investigation. Body mass index (BMI) is the most widely used marker for obesity and has been shown to correlate fairly with fat mass [39]. Epidemiological research has found compelling evidence showing that an elevated BMI increases CRC risk. The insulin signaling pathway has been put forward as a possible mechanism underlying the association between obesity and CRC, since increased levels of circulating insulin (as seen with increased fat tissue) stimulate colorectal epithelial cell proliferation in rat models [40].

Generally, stronger associations are observed in men than in women [41,42,43], and sex hormones seem to be the most probable explanation for this gender-based difference [44]. A prospective study [45] evaluating the relation between endogenous sex hormones and CRC risk found that in men, higher levels of testosterone and a lower ratio of estradiol over testosterone were associated with a decrease in the risk of developing CRC, even after controlling for BMI and C-peptide levels. The relative risks in the highest relative to the lowest quartile were 0.62 for testosterone (95% CI: 0.40–0.96) and 2.63 for the ratio (95% CI: 1.58–4.36) (*p* values for trend < 0.02) [45]. Furthermore, recent research has shown that elevated levels of endogenous estrogen are associated with a decreased risk of CRC in postmenopausal women, giving additional support to the protection conferred by estrogen in women [46]. In men, weight gain results in an increase in the level of estrogen, with a consequential reduction in testosterone and insulin resistance, which, in turn, increases the CRC risk [44].

Botma et al., in 2010, were the first to examine the association between obesity and colorectal adenomas in LS patients [47]. Tracking for 0 months, they shown that overweight (body mass index [BMI] over 25 kg/m^2^) increased the risk of incident colorectal adenomas in men living with LS (HR: 2.94; 95% CI: 1.11, 7.78) [47]. Specifically, and at baseline, overweight or obese men without adenoma were associated with an 8.7-fold risk of bowel adenoma, and an increase in BMI by 5 kg/m^2^ was associated with a higher risk of adenomas in a dose-dependent manner (HR: 1.84; 95% CI: 1.13, 3.02) [47]. Conversely, in women, a weight gain of more than 2 kg over almost two years was linked to an increased risk of adenoma diagnosis, but only if colorectal adenomas were already present [47]. In the CAPP2 study, among patients considered in the placebo arm, the risk of CRC was 2.4 times higher in obese patients with LS compared to those who were not obese. Here, the risk increased by 7% for every 1 kg/m^2^ increase in BMI, representing twice the excess risk for CRC seen in the general population [48]. Again, the greater CRC risk associated with each 1 kg/m^2^ increase in BMI appeared to be more expressive for men (HR: 1.12; 95% CI: 1.02 to 1.24; *p* = 0.02) than for women, though this difference was not statistically significant [48]. Campbell et al. examined the link between CRC and BMI by using data from a large population-based case–control study from Canada [49]. Men with a weight gain after the age of 20 were placed at a 72% increased risk of CRC (OR: 1.72; 95% CI: 1.22–2.41); this seemed clearer if the patient gained ≥21 kg and if theu had an already clinically defined familial risk of cancer (according to the Amsterdam or revised Bethesda criteria). Odds ratios were similar among subgroups of men without a familial risk of cancer. Lower categories of adulthood weight gain were not significantly associated with CRC risk. These effects were not reported in women [49].

Recently, Lazzeroni et al. [50] performed a meta-analysis of four studies reporting on obesity and CRC risk in LS patients, observing that obesity was strongly linked to an elevated risk of CRC in men (relative risk = 2.09; 95% CI = 1.23 to 3.55), but not in women (relative risk = 1.41; 95% CI = 0.46 to 4.27) [50]. However, the effect of BMI on CRC risk in pathogenic PMS2 and MSH6 carriers was not studied, revealing a potential limitation since adiposity could have been a decisive modifier considering the low penetrance of these two genes [50]. Indeed, it is already known that obesity induces chronic inflammation, which has negative effects on stem cells with acquired DNA damage, including those in the context of germline loss of MMR function, such as in LS [48]. However, beyond the known existence of sex differences in obesity-induced inflammation in metabolic and oncologic diseases [51,52], it seems that the additive effect of obesity on CRC risk might be counteracted in women by the effect of hormone exposure [50]. Therefore, a reasonable postulation for the lower CRC incidence in women may reside in the different capacity of different tissues/organs in handling hormone exposure. As a matter of fact, the estrogen signaling pathway mediated by ERβ has been shown to have anti-neoplastic effects in the colonic mucosa, such as altering immune surveillance, suppressing inflammatory pathways, and triggering apoptosis [53]. Lastly, it should also be stated that more than half of women with LS will experience a gynecologic malignancy as their primary cancer [54], thereby possibly influencing surveillance strategies and subsequent CRC incidence in women.

Regarding mutational status, Botma et al. [47] found a trend (though not significant) toward a greater excess of adenomatous polyps in overweight and obese patients with LS with germline mutation in MLH1 but not in MSH2 compared with those with BMI < 25 kg/m^2^ [47]. In the LS subgroup analysis of the CAPP2 study, MLH1 mutation and obesity was associated with a 3.72 times greater CRC risk (95% CI: 1.41 to 9.81), but no excess risk was observed in those with MSH2 or MSH6 mutation [48]. Likewise, a retrospective analysis of data from participants in the National Cancer Institute Colon Cancer Family Registry also found a significantly increased hazard ratio of CRC with increased BMI for MLH1 mutation carriers (HR: 1.36; 95% CI: 1.04 to 1.77) but not for those with mutation in MSH2, MSH6, or PMS2 [5]. It is striking to see an increased CRC risk due to adiposity affecting mostly those with pathogenic MLH1 variants. Concurrently, recent data proposed distinct pathways of CRC development in MLH1 vs. MSH2 carriers [55]. MLH1-associated CRCs may have a higher frequency of CTNNB1 mutations and MSH2-associated CRCs have a higher frequency of somatic APC mutations [55]. Nevertheless, since all MMR proteins are necessary for successful DNA repair, the true cause of the apparent greater susceptibility of MLH1 mutation carriers to the adverse effects of obesity remains unknown [48].

## 5. Behavioral Changes

Some studies aimed to look at lifestyle behaviors in LS to provide insights on how to advise this specific population regarding healthy lifestyle recommendations [56,57]. A study investigating diet-related health beliefs amongst individuals at a higher risk of cancers linked to HNPCC [23] found that most participants sustained a belief that cancer was linked to diet (76%) and to the possibility of a cancer risk mitigation through diet (83%). Men showed a lower likelihood than women of endorsing this relationship. A personal history of cancer and a high level of genetics knowledge were associated with the group supporting a diet–cancer connection [23]. In contrast, Visser et al. [58] discovered that a diagnosis of LS was not a key factor in following lifestyle recommendations but rather acted as a barrier to adopting a healthier lifestyle. Moreover, a small prospective study by Brouwer et al. [59] found scant evidence that a CRC diagnosis is associated with changes in dietary and lifestyle habits in persons with LS. In the study of Hoedjes et al. [60], acquired knowledge was a statistically significant determinant to the adherence to the recommendations on health behaviors (e.g., physical activity and red or processed meat intake), but not to the adherence to the body weight recommendation, which, sometimes, can be interpreted as the outcome of health behaviors. A personal cancer history was not associated with adherence to the recommendations of health-related choices. This conflicts with the notion of a putative window of opportunity, i.e., teachable moment, as is commonly described in the literature in relation to a lifestyle change following a cancer diagnosis [60]. However, in this study, the sample may not represent an LS population since study patients were more likely to be older, female, and to have been diagnosed with cancer, compared with those non-participants. In addition, a relatively high proportion of highly educated individuals was included, with the potential to limit the generalizability of the results. Nevertheless, some of the study’s findings underscore the importance of being knowledgeable about lifestyle recommendations, and proposes that fostering this knowledge is key to achieving adherence.

## 6. Final Discussion/Conclusions

The literature included and reviewed here (Table 2) has used a variety of study designs, mainly of a retrospective type, but also there were some with overlapping LS populations (such as the GeoLynch cohort and the Colon Family Register), which could have led to further limitations regarding the small populations studied, hence conditioning and restricting data availability independence and putatively decreasing the ability to adjust for potential confounding factors [9]. Moreover, retrospective case–control studies using interviews or self-reported questionnaires are prone to recall bias and to over- or under-reporting. In addition, the studies reported here concerned proven and/or suspected MMR gene mutation carriers, which, by including the latter, may also generate bias due to the probability of mutational mischaracterization [9]; henceforth, and for a better representation of MMR mutation burden, prospective studies should strive for genetic characterization. The limited available data suggest that certain lifestyle factors, namely diet, may play a role in altering the cancer risk conferred by the MMR mutations that cause LS. Obesity has an undoubtedly negative effect on general health and CRC risk in LS patients. It is well established that abdominal fat is acknowledged to have the most deleterious effects. Nonetheless, to date, no studies have investigated this association (through waist circumference, waist/hip, or even waist/height ratios) in relation to CRT in individuals harboring LS [47], warranting future studies to unravel this possible relation.

In summary, the available evidence suggests that people living with LS should follow general guidelines for diet and alcohol restriction and/or moderation; equally, they should thrive to achieve an overall health and quality of life to manage their CCR risk [57]. However, more research, preferentially of a prospective nature in well-designed clinical studies, is needed to better inform health and behavioral players and build recommendations for cancer prevention in LS patients.

## Figures and Tables

**Table 1:** Main dietary recommendations to prevent colorectal cancer.

Eat a diet rich in wholegrains, vegetables, fruit, and beans
Limit consumption of ‘fast food’ and other processed foods high in fat, starches, or sugars
Limit consumption of red and processed meat
Limit consumption of sugar-sweetened drinks
Limit alcohol consumption
Do not use supplements for cancer prevention

Adapted from World Cancer Research Fund/American Institute for Cancer Research. Diet, nutrition, physical activity and colorectal cancer: Continuous Update Project Expert Report. 2018 [8].

**Table 2:** Summary and characteristics of a selection of the studies included regarding the influence of diet, supplementation, and weight status in LS.

Author, Year	Type of Study	Sample	Investigation	Intervention	Results
Diet					
Voskuil DW et al. 2002 [17]	Retrospective case–control study	57 sporadic colorectal adenoma cases and 62 adenoma cases from LS families	Influence of meat consumption and preparation on sporadic and suspected LS–colorectal adenomas	-	Meat consumption is not a risk factor for adenoma formation in LS family members
Diergaarde B et al. 2007 [21]	Retrospective case–control study	145 cases and 103 controls	Association between dietary factors, cigarette smoking, and LS-associated CRT	-	Fruit consumption and dietary fiber intake might decrease the risk of colorectal tumors in LS
Kamiza AB et al. 2015 [30]	Retrospective cohort study	303 carriers of MLH1 or MSH2 mutations	Risk factors associated with CRC in patients with MLH1 and MSH2 germline mutations	-	High-level fruit intake was associated with a decreased CRC risk/MSH2 carriers, and alcohol intake showed a twofold increase in CRC risk
Mathers JC et al. 2022 [13]	RCT	463 LS participants received RS and 455 LS participants in placebo arm	Long-term effects of resistant starch on cancer incidence in patients with LS	30 g RS daily or placebo for up to 4 years	RS reduces morbidity associated with extracolonic cancers, especially upper GI cancers
Dashti SG et al. 2017 [25]	Retrospective cohort study	1925 LS individuals	Association between lifetime alcohol consumption and CRC risk for MMR gene mutation	-	Alcohol consumption (particularly more than 28 g/day) is associated with an increased CRC risk in patients with LS
Miguchi M et al. 2018 [29]	Retrospective cohort study	66 LS individuals	Alcohol and the risk of early-onset CRC in Japanese patients with LS	-	Alcohol was significantly correlated with an increased risk of early-onset CRC
Supplementation					
Cats A et al. 1995 [24]	RCT	30 subjects (first-degree family members of patients with LS)	Effect of supplemental oral calcium on epithelial cell proliferation in the distal colorectum in patients at a high risk of developing LS–colorectal cancer	Oral calcium carbonate supplements (1.5 g) or placebo 3 times a day during a 12-week period	Oral calcium supplementation caused a minor non statistically significant reduction in epithelial cell proliferation in the rectum and no effect in the sigmoid and descending colon; calcium probably has no value in the prevention of CRC in LS
Heine-Bröring RC et al. 2013 [18]	Prospective cohort study	470 individuals with LS	Associations between dietary supplement use and colorectal adenoma risk	-	No inverse associations between dietary supplement use and occurrence of colorectal adenomas among individuals with LS
Jung AY et al. 2014 [19]	Prospective cohort study	470 individuals with LS	Associations between dietary intakes of folate; vitamins B2, B6, and B12; and methionine and CRT development	-	No association between intake of any dietary B vitamin or methionine and CRT development among individuals with LS
Chau R et al. 2016 [20]	Prospective cohort study	1966 LS individuals	Associations between self-reported multivitamin, calcium, and folic acid supplement intake and CRC risk	-	Multivitamin and calcium supplements might be associated with a decreased risk of CRC in LS
Weight status					
Campbell PT et al. 2007 [49]	Retrospective case–control study	2696 cases (familial risk based on a family history indicative of LS) and 2668 controls	Link between CRC and BMI at two reference periods (BMI 2 years prior and BMI at age 20 years); weight gain since age 20 years; and height	-	Obesity is associated with an increased risk of CRC in the general population and in individuals with a family history indicative of LS
Botma A et al. 2010 [47]	Prospective cohort study	486 individuals with LS	BMI and colorectal adenoma occurrence in persons with LS	-	Excess body weight increased the risk of incident colorectal adenomas in men with LS
Movahedi M et al. 2015 [48]	Prospective cohort study	937 individuals with LS	Association between BMI and cancer risk in patients with LS	-	Obesity is associated with increased CRC risk in patients with LS

Abbreviations—BMI: body mass index; CRC: colorectal cancer; CRT: colorectal tumors; MMR: mismatch repair; LS: Lynch syndrome; RCT: randomized controlled trial; RS: resistant starch.
